# Increasing the activities of protective enzymes is an important strategy to improve resistance in cucumber to powdery mildew disease and melon aphid under different infection/infestation patterns

**DOI:** 10.3389/fpls.2022.950538

**Published:** 2022-08-17

**Authors:** Quancheng Zhang, Menghan Zhou, Jungang Wang

**Affiliations:** College of Agriculture, Shihezi University, Shihezi, China

**Keywords:** *Cucumis sativa* L., *Sphaerotheca fuliginea* (Schlecht.) Poll., *Aphis gossypii* Glover, photosynthesis, protective enzymes, metabolism, resistance

## Abstract

Powdery mildew, caused by *Sphaerotheca fuliginea* (Schlecht.) Poll., and melon aphids (*Aphis gossypii* Glover) are a typical disease and insect pest, respectively, that affect cucumber production. Powdery mildew and melon aphid often occur together in greenhouse production, resulting in a reduction in cucumber yield. At present there are no reports on the physiological and biochemical effects of the combined disease and pest infection/infestation on cucumber. This study explored how cucumbers can regulate photosynthesis, protective enzyme activity, and basic metabolism to resist the fungal disease and aphids. After powdery mildew infection, the chlorophyll and free proline contents in cucumber leaves decreased, while the activities of POD (peroxidase) and SOD (superoxide dismutase) and the soluble protein and MDA (malondialdehyde) contents increased. Cucumber plants resist aphid attack by increasing the rates of photosynthesis and basal metabolism, and also by increasing the activities of protective enzymes. The combination of powdery mildew infection and aphid infestation reduced photosynthesis and basal metabolism in cucumber plants, although the activities of several protective enzymes increased. Aphid attack after powdery mildew infection or powdery mildew infection after aphid attack had the opposite effect on photosynthesis, protective enzyme activity, and basal metabolism regulation. Azoxystrobin and imidacloprid increased the contents of chlorophyll, free proline, and soluble protein, increased SOD activity, and decreased the MDA content in cucumber leaves. However, these compounds had the opposite effect on the soluble sugar content and POD and CAT (catalase) activities. The mixed ratio of the two single agents could improve the resistance of cucumber to the combined infection of powdery mildew and aphids. These results show that cucumber can enhance its pest/pathogen resistance by changing physiological metabolism when exposed to a complex infection system of pathogenic microorganisms and insect pests.

## Introduction

Cucumber (*Cucumis sativa* L.) is a worldwide economic crop, and greenhouse culture can maintain cucumber production throughout the year. Cucumber production is often affected by a phytophagous insects and pathogenic microorganisms, resulting in a reduction in the yield of cucumber fruits (Bagi et al., 2014; Bian et al., 2020). In greenhouses and fields, powdery mildew disease caused by *Sphaerotheca fuliginea* (Schlecht.) Poll. and the melon aphid (*Aphis gossypii* Glover) are important biotic factors that threaten cucumber production (Berg et al., 2015; Qi et al., 2020). These two often attack individually or together, and they can alternate. Under different infection pressures, plants will adopt specific resistance strategies to alleviate stress and improve adaptability (Zhou et al., 2013; Cui et al., 2015). However, it is unclear how cucumber plants regulate and change their physiological responses to powdery mildew and melon aphid under different infection patterns.

In our review of the published literature, we found that only a few studies reported the interactions between cucumber and powdery mildew and cucumber and melon aphids (Arauz et al., 2010; Gao et al., 2017; Zhang H. M. et al., 2021). In the cucumber-powdery mildew system, early studies showed that cucumber plants were able to resist infection by the powdery mildew pathogen by regulating the metabolism and synthesis of chlorophyll and osmoregulatory substance (Wang H. Z. et al., 2006; Jiang and Si, 2010). Several recent studies have described the epidemiology, host specificity, and genome of cucumber powdery mildew pathogens (Berg et al., 2015; Zhang P. et al., 2021). In the cucumber-melon aphid system, cucumber plants resist damage from melon aphids by regulating the activities of peroxidase (POD), polyphenol oxidase (PPO), and phenylalanine ammonia lyase (PAL) (Gao et al., 2017). Our previous research found that cucumber plants resist powdery mildew infection and melon aphid colonization by regulating photosynthesis, basic metabolite levels, and the activities of protective enzymes (Zhou et al., 2013; Cui et al., 2015). However, these studies revealed some physiological regulation strategies of cucumber against powdery mildew and melon aphids. We should note that in the field and the greenhouse, powdery mildew and melon aphid showed more complex and alternating infection patterns. At present, there is no report on the physiological regulation strategies of cucumber under the combination of powdery mildew infection and melon aphid infestation.

Several recent reports have systematically studied the relationships among pathogens, insects, and host plants (Gross et al., 2021; van Dijk et al., 2021; Franco et al., 2022; Laihonen et al., 2022). Of these, research on insect-virus-host plant systems is the most extensive (Ziebell et al., 2011; Casteel et al., 2015; Flasco et al., 2021; Wang Y. F. et al., 2021). In these studies, the host can resist the infection of pathogens and insect vector by adjusting its metabolism, including photosynthesis, glycolysis, and respiration (Spyropoulou et al., 2014; Groen et al., 2016; Ye et al., 2017). At present, there are very few reports describing the fungal pathogen-phytophagous insect-host plant system (Perkins et al., 1996; Meng, 2013; Franco et al., 2022). An early study showed that aphid feeding stimulated peroxidase activity during the infection of watermelon by *Colletotrichum gloeosporioides* (Perkins et al., 1996), and a more recent study showed that *Colletotrichum falcatum* infection regulates the olfactory behavior of the sugarcane borer (*Diatraea saccharalis*) to increase its own fitness (Franco et al., 2022). Several other studies showed that there are differences in the defense regulation of cucumber against the leaf miner *Liriomyza huidobrensis* and cucumber downy mildew (*Pseudoperonos poracubensis*) in the cucumber-*L. huidobrensis*-cucumber downy mildew system (Meng et al., 2012, 2014; Sun et al., 2012, 2013). We also found a similar phenomenon in the cucumber-powdery mildew-aphid system (Zhou et al., 2013; Cui et al., 2015). However, it is unclear whether there are differences in the physiological defense response regulation mechanisms of cucumber under different infection modes of melon aphid and powdery mildew.

Here, based on previous studies, we further explored how cucumber plants respond to different infection modes of the powdery mildew pathogen and melon aphid by regulating physiological mechanisms. We hypothesized that cucumber can respond to different infection modes of powdery mildew and melon aphid by regulating photosynthesis, protective enzyme activities, and basic metabolism (Zhou et al., 2013; Cui et al., 2015). However, we do not know how these adjustment strategies change. Therefore, the objectives of this study were to explore (1) the physiological regulation strategy of cucumber plants in response to infection by the powdery mildew fungus *Sphaerotheca fuliginea*, (2) the physiological regulation strategy of cucumber plants in response to melon aphid infestation, (3) the physiological regulation strategy of cucumber in response to the combination of powdery mildew disease and melon aphid infestation, (4) the physiological regulation strategy of cucumber in response to different degrees of powdery mildew infection and melon aphid infestation, and (5) the physiological regulation strategy of cucumber in response to powdery mildew infection and aphid feeding under pesticide application. By analyzing the changes that occurred in the physiological responses of cucumber plants to the different infection modes of powdery mildew and melon aphid, we were able to clarify the differences in defense response strategies of the cucumber host in the cucumber-powdery mildew-melon aphid system. The results of this study will provide a reference for identifying and understanding the changes that occur in the physiological regulation strategies of plants in response to the combination of pathogen infection and insect pest infestation.

## Materials and methods

### Plants

Cucumber variety “Xinjinyan No.4” was provided by the College of Agriculture, at Shihezi University. Cucumber seeds were wrapped in black cloth and soaked in a beaker containing water at 40°C for 6 h. The water was then removed and the seeds were transferred to a Petri dish where they were allowed to germinate for 24 h in an incubator at 40°C. When the bud lengths were 3–4 mm, the seeds were planted in plastic cups with holes in the bottom containing sterile seedling substrate. The diameter of the cups was 9 cm, and there was one cucumber seedling per cup. When the cotyledons were fully expanded and the first true leaf had developed, the seedlings were used for aphid feeding and pathogen inoculation.

### Aphids and fungal pathogen

Melon aphids (*A. gossypii*) were originally collected from cucumber leaves in the field, and an artificial breeding population was established and maintained in the insect greenhouse of Shihezi University. Over 30 generations of aphids have been subcultured on cucumber plants. Feeding temperature was 26 ± 1°C, relative humidity was 60–80%, and the light intensity was 9,000 lx with a 14 h light/10 h dark photoperiod.

The powdery mildew fungus (*S. fuliginea*) was collected from fresh diseased leaves of cucumber in the field, and a single, typical powdery mildew lesion that was well-isolated by distance from the other lesion spots excised. After dipping in water, the lesion spots were gently attached to the adaxial surface of cultured cucumber cotyledons to propagate the strains. The culture temperature was 20 ± 0.5°C, relative humidity was 60–80%, and the light intensity was 9,000 lx with a 16 h light/8 h dark photoperiod.

### Chemicals

Ten percent imidacloprid WP was provided by Jiangsu Limin Chemical Co., Ltd. (Jiangsu, China). 25% azoxystrobin SC was provided by Xianzhengda (Qingdao) Crop Protection Co., Ltd. (Shandong, China). Sodium dodecyl sulfate (SDS) was provided by Xilong Scientific Co., Ltd. (Guangdong, China).

### Preparation of powdery mildew spore suspension

Cucumber cotyledons with fresh powdery mildew lesions were removed and placed in a solution of SDS (1 mg/ml) to wash off the conidia. When the concentration of suspended spores was 30–40 per field of vision under 15 × 10 times of microscope, it could be used in the inoculation test.

### Powdery mildew infection on chlorophyll content, protective enzyme activity, and nutrient metabolism in cucumber leaves

Cucumber seeds were planted in flowerpots with a diameter of 9 cm in the greenhouse and grown normally under natural conditions. When the first true leaves of the cucumber seedlings reached 10 cm^2^ (determination of leaf area by YMJ-G handheld leaf area measuring instrument), the fungal spore suspension was used to inoculate the cucumber leaves by the smearing method (Jia et al., 2006). The chlorophyll content, activities of SOD (superoxide dismutase), POD (peroxidase), and CAT (catalase), and the soluble protein, soluble sugar, free proline, and MDA (malondialdehyde) contents of cucumber leaves were measured at 1, 3, 5, 7, and 9 days after inoculation. We used uninoculated cucumber seedlings as the controls. Each treatment group and control group consisted of five cucumber seedlings, and each treatment was repeated three times.

### Aphid infestation at different densities on chlorophyll content, protective enzyme activity, and nutritional metabolism in cucumber leaves

Cucumber plants were grown in flowerpots (9-cm diameter) in the greenhouse under natural conditions. When the first true leaves reached 10 cm^2^, aphids (newly born wingless nymphs) were transferred onto the cucumber seedlings at different densities in the greenhouse and covered with nylon net. We used five treatments at different densities; 160 aphids/leaf (T1), 80 aphids/leaf (T2), 40 aphids/leaf (T3), 20 aphids/leaf (T4), and 0 aphids (CK). After the aphids were allowed to feed on the seedlings for 48 h, the leaves were collected for the assays. Each treatment group and control group consisted of five cucumber seedlings, and each treatment was repeated three times.

### Aphid infestation on chlorophyll content, protective enzyme activity, and nutrient metabolism in cucumber leaves after powdery mildew infection

Cucumbers were planted in greenhouse at the same time in the flowerpot of 9 cm in diameter and grew under natural conditions. When the first true leaf of cucumber seedlings reached 10 cm^2^, the fungal spore suspension was inoculated on cucumber leaves by smearing method (Jia et al., 2006). When the lesion area on the equal leaf surface was 5–10%, the plants with the same disease index were selected, and aphids (newly born nymphs of wingless aphids) with different population densities were inoculated onto cucumber seedlings in the greenhouse and meshed. We set five population densities, namely 160 aphids (T1), 80 aphids (T2), 40 aphids (T3), 20 aphids (T4), and 0 aphids (CK). Set uninfected controls. After aphids were fed on the above for 48 h, the leaves were collected for determination. Each treatment group and control group were taken five melon seedlings, each treatment repeated three times.

### Time series of powdery mildew infection and aphid feeding on chlorophyll content, protective enzyme activity, and nutrient metabolism in cucumber leaves

The cucumber seedlings were grown in the greenhouse and were inoculated when the first leaves reached 10 cm^2^ as described for the previous experiments. Test 1 (T1): The fungal spore suspension was inoculated onto the cucumber seedlings in the greenhouse, and 100 aphids were placed on the leaves when the leaf lesion area reached 5–10%. After 48 h, the leaves were collected for the assays. Test 2 (T2): The cucumber seedlings were inoculated with the fungal spore suspension in the greenhouse, allowed to dry naturally, and 100 aphids were then placed on the leaves. After 48 h, the leaves were collected for the assays. Test 3 (T3): 100 aphids were placed on the cucumber seedlings in the greenhouse. After 48 h of feeding, the leaves were inoculated with fungal spore suspension. When the leaf lesion area reached 5–10%, the leaves were collected for the assays. Each treatment group and control group consisted of five cucumber seedlings, and each treatment was repeated three times.

### Combined powdery mildew infection and aphid infestation on chlorophyll content, protective enzyme activity, and nutrient metabolism in cucumber leaves under pesticide application

Based on the recommended application rates of the pesticides, four concentrations of azoxystrobin were used: 4 mg L^–1^ (A1), 2 mg L^–1^ (A2), 1 mg L^–1^ (A3), and 0 mg L^–1^ (A4). The four concentrations of imidacloprid were: 750 mg L^–1^ (B1), 500 mg L^–1^ (B2), 250 mg L^–1^ (B3), and 0 mg L^–1^ (B4). Azoxystrobin and imidacloprid at different concentrations were mixed in pairs. When the first true leaves of the cucumber seedlings grown in the greenhouse reached 10 cm^2^, the prepared powdery mildew spore suspension was sprayed on the cucumber leaves, and then 100 aphids were transferred with a soft brush onto the cucumber seedling leaves. When the powdery mildew lesion areas reached 25%, the azoxystrobin and imidacloprid mixtures were applied by spraying, and the leaves were collected after 48 h. Each treatment group and control group had five cucumbers seedlings, and each treatment was repeated three times.

### Determination of chlorophyll content, protective enzyme activity, and nutrient contents in cucumber leaves

Chlorophyll was extracted from the leaf tissue with alcohol as described by Arnon (1949). 0.1 g cucumber leaves were cut, then added with 10 mL mixed solution of acetone and ethanol (V_acetone_: V _ethanol_ = 1: 1), and soaked in dark environment until the leaves became white. The absorbance values of chlorophyll a and chlorophyll b were measured at 645 and 663 nm, respectively. Chlorophyll content = (20.29 OD_645_ + 8.04 OD_663_) × V/1,000 W, V is the volume of extraction liquid, and W is the weighed leaf mass.

Soluble protein contents were determined using the G-250 dye colorimetric method (Bradford, 1976). 0.5 g cucumber leaves were weighed, add 5 mL distilled water, ice bath grinding, 5,000 r min^–1^ centrifugal 10 min, draw supernatant, placed in 10 mL centrifuge tube. 2 mL of distilled water was added to the residual residue, and centrifuged again. The supernatant was taken and mixed with the first supernatant. 0.5 mL supernatant was mixed with 5 mL G-250 dye, and the absorbance was measured at 595 nm.

Soluble sugar contents were determined by the anthrone method (Dreywood, 1946). 0.5 g cucumber leaves were put into a 10 mL graduated centrifuge tube, added 4 mL of 80% ethanol, and boiled in water at 80°C for 30 min with constant shaking. After a centrifugation at 3,000 r min^–1^ for 10 min, the supernatant was collected and placed in a 10 ml test tube. The residue was repeated the above process, and the supernatant was collected. By mixing 1 ml of supernatant and 4 mL of anthrone reagent, the mixture was water bathed in boiling water for 10 min. After cooling, the sample was performed colorimetry at 625 nm.

Free proline contents were determined by the acid ninhydrin method (Bates et al., 1973). 0.5 g cucumber leaves were placed in a mortar, 5 mL of 3% sulfosalicylic acid was added and then the sample was grinded, homogenized and then transferred to a centrifuge tube, extracted in a boiling water bath for 10 min and cooled to room temperature. After centrifugation at 3,000 r⋅min^–1^ for 10 min, 2 mL supernatant was mixed with 4 mL of acid ninhydrin reagent, 2 mL of glacial acetic acid and 2 mL of 3% sulfosalicylic acid, and the reaction mixture was placed in a boiling water bath for 1 h. After cooling to room temperature, 4 ml of toluene was added. After sufficient shaking, the red toluene was placed in a cuvette, and the absorbance was measured at 520 nm.

Malondialdehyde contents were determined by the thiobarbituric acid method (Heath and Packer, 1968), the maximum absorption peak appears at 532 nm.

0.5 g cucumber leaves were weighed, then put into the tissue homogenater containing 2.5 mL 50 mmol L^–1^ pH = 7.8 phosphate buffer. The homogenate was fully grinded in ice water bath. The homogenate was transferred into 5 mL centrifuge tube, and the volume was fixed to 5 mL with phosphate buffer. The homogenate was centrifuged at 10,000 r min^–1^ and 4°C for 10 min, and the supernatant was extracted as enzyme source solution. POD activity was determined by the guaiacol method (Zaharieva et al., 1999). The activity of SOD was determined by the inhibition photoreduction method, where the amount of the enzyme required to inhibit NBT reduction by 50% is defined as one unit of enzyme activity (Giannopolities and Ries, 1977). CAT activity was determined by the UV absorption method (Chance and Maehly, 1955). All of the enzymes were retest at least five times per treatment, and the average value was taken.

### Statistical analyses

Microsoft Excel 2010 was used for data processing and plotting. SPSS 20.0 data processing software was used for statistics, and the mean values and standard errors were calculated. The LSD test and t-test were used to test the significance of the differences in the means.

## Results

### Powdery mildew infection on chlorophyll content, protective enzyme activity, and nutrient metabolism in cucumber leaves

**FIGURE 1:**
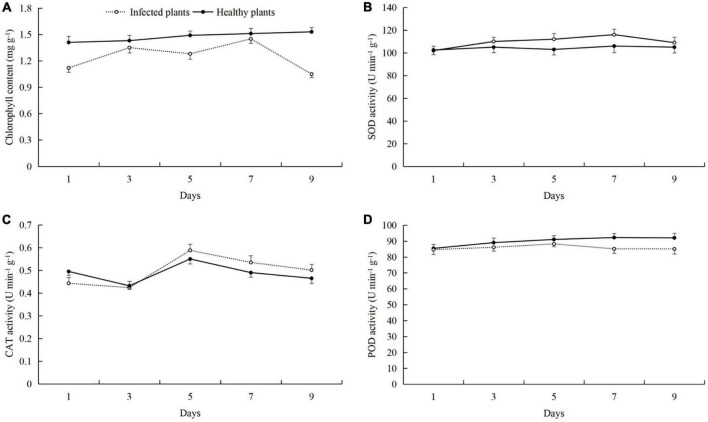
Effects of powdery mildew infection on **(A)** chlorophyll content and the activities of **(B)** superoxide dismutase (SOD), **(C)** catalase (CAT), and **(D)** peroxidase (POD) in cucumber leaves.

After powdery mildew infection of cucumber leaves, we found that the leaf chlorophyll content was reduced, and the activities of SOD, CAT, and POD were increased (Figure 1). Compared with healthy plants, the chlorophyll contents of cucumber plants that were infected to powdery mildew disease on day 9 after inoculation were significantly decreased by 0.48 mg g^–1^, and the healthy plants were 1.06 mg g^–1^ (*t*-test: *t* = 6.30; *d.f.* = 14; *p* = 0.026; Figure 1A). SOD activity in the infected plants was highest on day 7 (113.68 U min^–1^ g^–1^), and the healthy plants were 149.33 U min^–1^ g^–1^ (Figure 1B). CAT activity was the highest on day 5 (0.58 U min^–1^ g^–1^), and the healthy plants were 0.55 U min^–1^ g^–1^ (Figure 1C). POD activity was the highest on day 9 after inoculation (94.07 U min^–1^ g^–1^), and the healthy plants were 85.24 U min^–1^ g^–1^ (Figure 1D).

**FIGURE 2:**
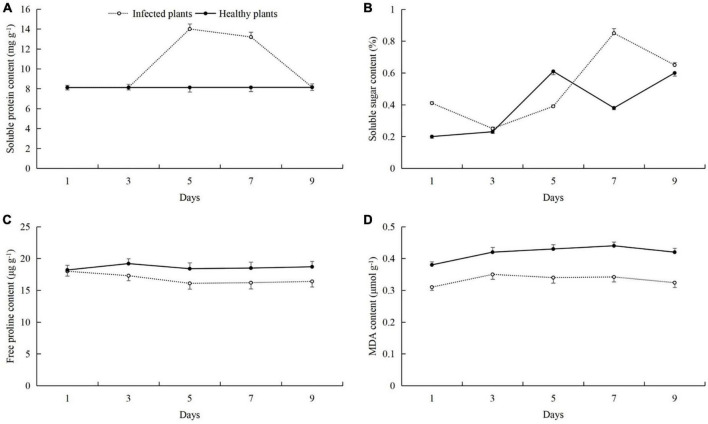
Effects of powdery mildew infection on the contents of **(A)** soluble proteins, **(B)** soluble sugars, **(C)** free proline, and **(D)** malondialdehyde (MDA) in cucumber leaves.

After the cucumber leaves were infected with powdery mildew disease, the contents of soluble proteins, soluble sugars, and MDA were increased, and the free proline content was decreased (Figure 2). The soluble protein content of infected plants was the highest on day 5, and was 5.83 mg g^–1^ higher than in healthy plants (8.17 mg g^–1^) (*t*-test: *t* = 4.15; *d.f.* = 14; *p* = 0.03; Figure 2A). The contents of soluble sugars and MDA were the highest on day 7, and were higher than in healthy plants by 0.47% (*t*-test: *t* = 2.39; *d.f.* = 14; *p* = 0.001; Figure 2B) and 0.12 μmol g^–1^ (*t*-test: *t* = 2.02; *d.f.* = 14; *p* = 0.025; Figure 2D), respectively. The free proline content in the infected plants was the highest at day 1, and was 18.44 μg g^–1^ (Figure 2C).

### Aphid feeding at different densities on chlorophyll content, protective enzyme activity, and nutritional metabolism in cucumber leaves

**FIGURE 3:**
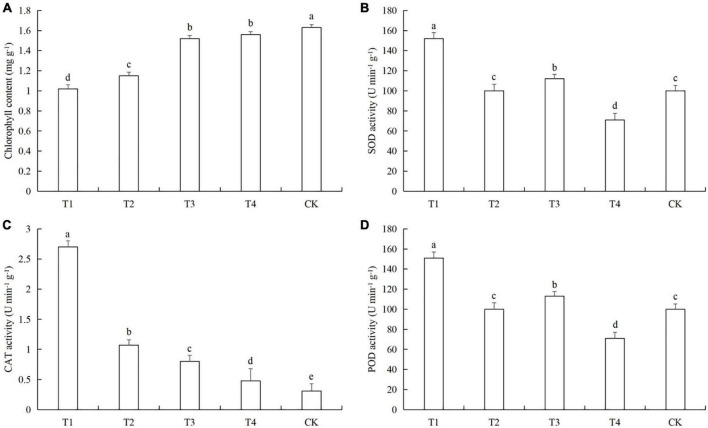
Effects of different aphid densities on **(A)** chlorophyll content and the activities of **(B)** superoxide dismutase (SOD), **(C)** catalase (CAT), and **(D)** peroxidase (POD) in cucumber leaves. T1, 160 aphids; T2, 80 aphids; T3, 40 aphids; T4, 20 aphids; CK, 0 aphids. The mean value ± *p* < 0.05 error bar data were presented in the figure and different alphabetical letters indicate the significant differences between the aphid density treatments.

Aphids feeding caused a significant decreased in chlorophyll content in cucumber leaves at all tested densities. CAT activity was significantly increased in the four aphid treatments. The activities of SOD and POD were very similar in the five treatments, but the pattern was very different from CAT (Figure 3). The chlorophyll contents in treatments T1 was lower than in the control by 37.2% (LSD test: *p* = 0.025) (Figure 3A). The SOD activity in T1 was higher than in the control by 25.4% (LSD test: *p* = 0.022) (Figure 3B). The increase in CAT activity in T1 compared to the control were 697.1% (LSD test: *p* = 0.001) (Figure 3C). The POD activities in T1 and T3 were higher than in the control by 54.2% (LSD test: *p* = 0.027) and 14.3% (LSD test: *p* = 0.031), respectively (Figure 3D). Aphid feeding at low density reduced the SOD and POD activities in cucumber leaves, while aphid feeding at high density caused an increase in the SOD and POD activities in cucumber leaves.

**FIGURE 4:**
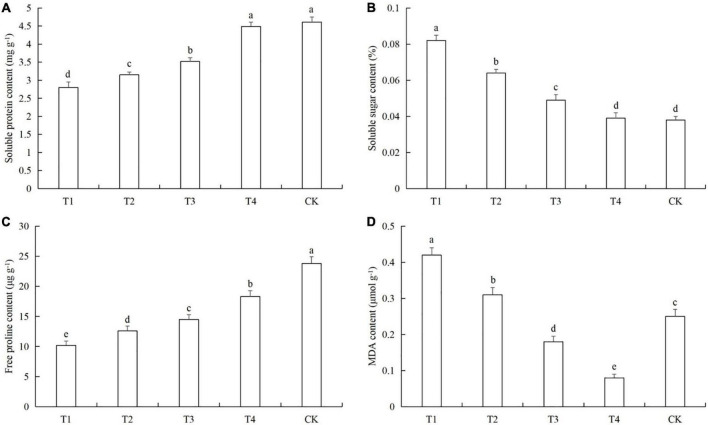
Effects of the different aphid density treatments on the contents of **(A)** soluble proteins, **(B)** soluble sugars, **(C)** free proline, and **(D)** malondialdehyde (MDA) in cucumber leaves. T1, 160 aphids; T2, 80 aphids; T3, 40 aphids; T4, 20 aphids; CK, 0 aphids. The mean value ± *p* < 0.05 error bar data were presented in the figure and different alphabetical letters indicate the significant differences between the aphid density treatments.

After aphid feeding, the contents of soluble protein and free proline in cucumber leaves decreased significantly, while the content of soluble sugars increased significantly. Low density aphid feeding resulted in decreased MDA content, and aphid feeding at high density increased the MDA content (Figure 4). The soluble protein contents in the T1 treatments were 38.4% (LSD test: *p* = 0.028) (Figure 4A). The soluble sugar contents in T1 was 127.8% (LSD test: *p* = 0.005) higher than in the control (Figure 4B). The free proline contents in T1 was 56.4% (LSD test: *p* = 0.018) lower than in the control (Figure 4C). The MDA contents of T1 and T2 were 68.2% (LSD test: *p* = 0.013) and 24.4% (LSD test: *p* = 0.029) higher than in the control, respectively (Figure 4D).

### The combined effects of aphid feeding and powdery mildew infection on chlorophyll content, protective enzyme activity, and nutrient metabolism in cucumber leaves

**FIGURE 5:**
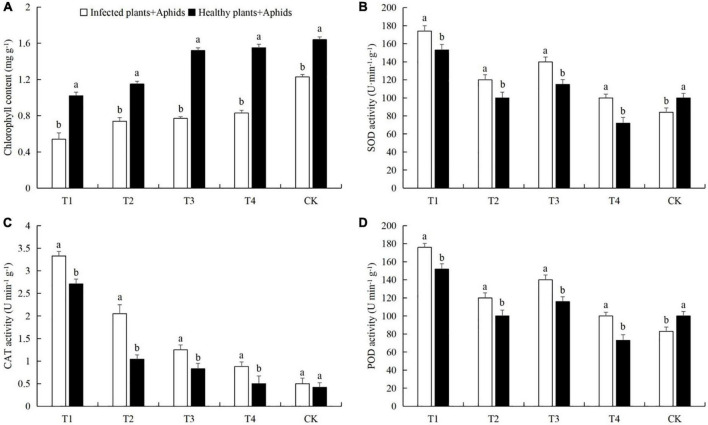
Effects of different aphid densities on **(A)** chlorophyll content and the activities of **(B)** superoxide dismutase (SOD), **(C)** catalase (CAT), and **(D)** peroxidase (POD) in cucumber leaves after powdery mildew infection. T1, 160 aphids; T2, 80 aphids; T3, 40 aphids; T4, 20 aphids; CK, 0 aphids. The mean value ± *p* < 0.05 error bar data were presented in the figure and different alphabetical letters indicate the significant differences between the infected plants + aphids and the healthy plants + aphids.

**FIGURE 6:**
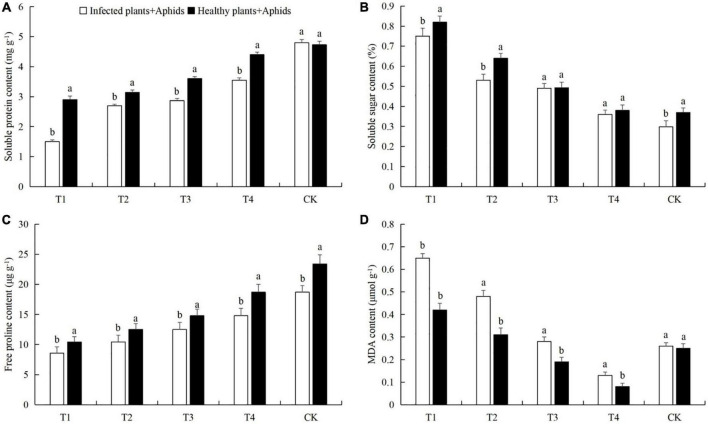
Effects of different aphid densities on the contents of **(A)** soluble proteins, **(B)** soluble sugars, **(C)** free proline, and **(D)** malondialdehyde (MDA) in cucumber leaves after powdery mildew infection. T1, 160 aphids; T2, 80 aphids; T3, 40 aphids; T4, 20 aphids; CK, 0 aphids. The mean value ± *p* <0.05 error bar data were presented in the figure and different alphabetical letters indicate the significant differences between the infected plants + aphids and the healthy plants + aphids.

The combined effects of aphid feeding and powdery mildew infection reduced the contents of chlorophyll, soluble protein, soluble sugars, and free proline in cucumber leaves, and increased the activities of the three protective enzymes and the MDA content (Figures 5, 6). At a density of 160 aphids/leaf, the chlorophyll, soluble protein, and free proline contents in the leaves subjected to both pathogen infection and aphid feeding were the lowest, and the protective enzyme activities, soluble sugar content, and MDA content were the highest (Figures 5, 6). Also, compared with healthy plants + aphids, the contents of chlorophyll and soluble protein in infected plants + aphids were significantly decreased 49.02% (*t*-test: *t* = 1.58; *d.f.* = 14; *p* = 0.018; Figure 5A), 48.28% (*t*-test: *t* = 2.44; *d.f.* = 14; *p* = 0.025; Figure 6A), respectively. The SOD, CAT, and POD activities and the contents of MDA were significantly increased 13.73% (*t*-test: *t* = 2.52; *d.f.* = 14; *p* = 0.043; Figure 5B), 15.79% (*t*-test: *t* = 2.30; *d.f.* = 14; *p* = 0.029; Figure 5C), 24.35% (*t*-test: *t* = 2.06; *d.f.* = 14; *p* = 0.030; Figure 5D), and 35.38% (*t*-test: *t* = 2.37; *d.f.* = 14; *p* = 0.033; Figure 6D), respectively.

### Time series of powdery mildew infection and aphid feeding on chlorophyll content, protective enzyme activity, and nutrient metabolism in cucumber leaves

**FIGURE 7:**
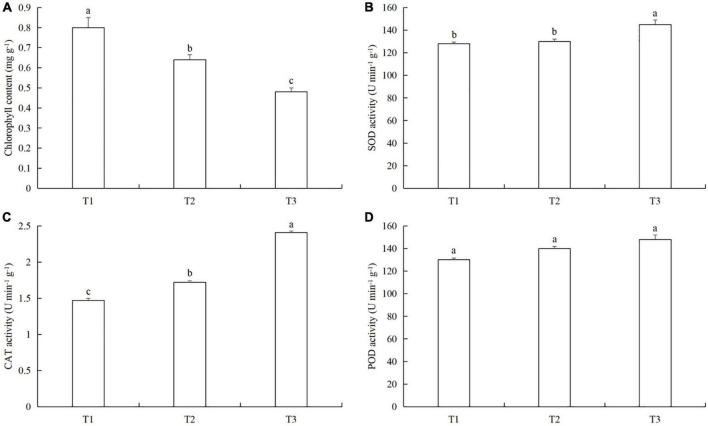
The effects of timing and order of powdery mildew infection and aphid colonization on **(A)** chlorophyll content and the activities of **(B)** superoxide dismutase (SOD), **(C)** catalase (CAT), and **(D)** peroxidase (POD) in cucumber leaves. T1, Powdery mildew followed by aphids. T2, Simultaneous powdery mildew infection and aphid colonization. T3, Aphid colonization followed by powdery mildew infection. The mean value ± *p* < 0.05 error bar data were presented in the figure and different alphabetical letters indicate the significant differences between the different time treatments.

The chlorophyll content in T1 was 20.7% (LSD test: *p* = 0.023) and 41.7% (LSD test: *p* = 0.026) higher than in T2 and T3, respectively (Figure 7A). The SOD activity in T3 was 10.3% (LSD test: *p* = 0.013) and 13.0% (LSD test: *p* = 0.01) higher than in T2 and T1, respectively (Figure 7B). The CAT activity in T3 was 26.9% (LSD test: *p* = 0.031) and 39.1% (LSD test: *p* = 0.028) higher than in T2 and T1, respectively (Figure 7C). There were no significant differences in POD activity between T1, T2, and T3 (Figure 7D).

**FIGURE 8:**
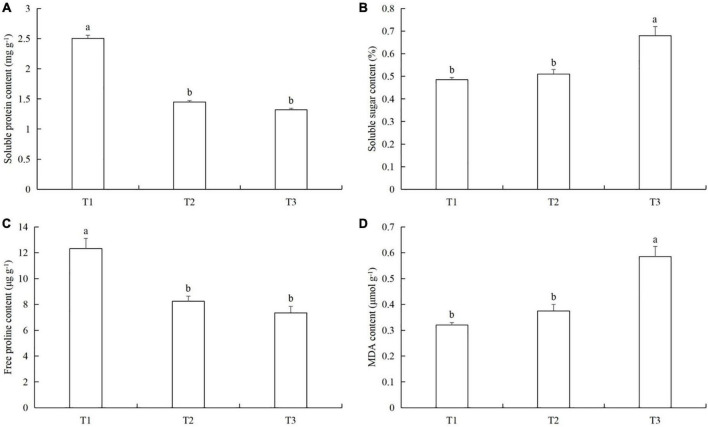
The effects of timing and order of powdery mildew infection and aphid colonization on the contents of **(A)** soluble proteins, **(B)** soluble sugars, **(C)** free proline, and **(D)** malondialdehyde (MDA) in cucumber leaves. T1, Powdery mildew followed by aphids. T2, Simultaneous powdery mildew infection and aphid colonization. T3, Aphid colonization followed by powdery mildew infection. The mean value ± *p* < 0.05 error bar data were presented in the figure and different alphabetical letters indicate the significant differences between the different time treatments.

The soluble protein content in T1 was 41.9% (LSD test: *p* = 0.019) and 47.8% (LSD test: *p* = 0.022) higher than in T2 and T3, respectively (Figure 8A). The soluble sugar content in T3 was 26.1% (LSD test: *p* = 0.031) and 28.9% (LSD test: *p* = 0.037) higher than in T2 and T1, respectively (Figure 8B). The free proline content in T1 was 33.2% (LSD test: *p* = 0.025) and 40.7% (LSD test: *p* = 0.022) higher than in T2 and T3, respectively (Figure 8C). The MDA content in T3 was 36.2% (LSD test: *p* = 0.02) and 44.8% (LSD test: *p* = 0.015) higher than in T2 and T1, respectively (Figure 8D).

### Combined powdery mildew infection and aphid feeding on chlorophyll content, protective enzyme activity, and nutrient metabolism in cucumber leaves under pesticide application

**FIGURE 9:**
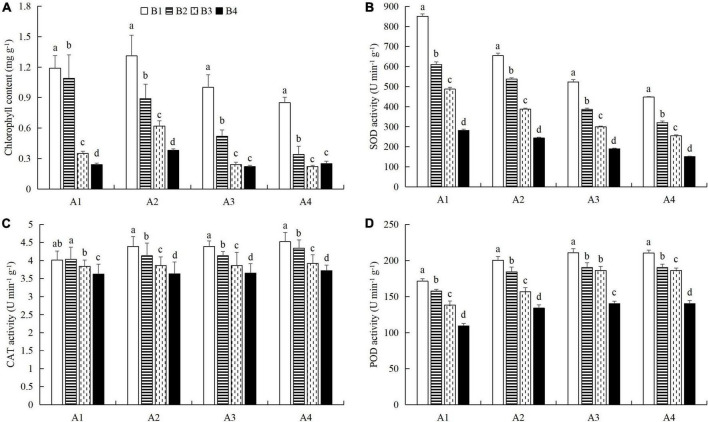
The effects of imidacloprid on **(A)** chlorophyll content and the activities of **(B)** superoxide dismutase (SOD), **(C)** catalase (CAT), and **(D)** peroxidase (POD) in cucumber leaves at four azoxystrobin concentrations. Azoxystrobin concentrations: 4 mg L^–1^ (A1), 2 mg L^–1^ (A2), 1 mg L^–1^ (A3), and 0 mg L^–1^ (A4). Imidacloprid concentrations: 750 mg L^–1^ (B1), 500 mg L^–1^ (B2), 250 mg L^–1^ (B3), and 0 mg L^–1^ (A4). The mean value ± *p* < 0.05 error bar data were presented in the figure and different alphabetical letters indicate the significant differences between the different imidacloprid concentrations.

**FIGURE 10:**
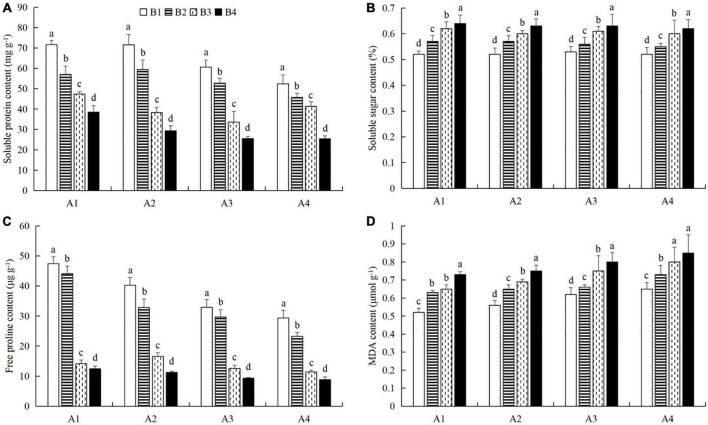
The effects of imidacloprid on the contents of **(A)** soluble proteins, **(B)** soluble sugars, **(C)** free proline, and **(D)** malondialdehyde (MDA) in cucumber leaves at different azoxystrobin concentrations. Azoxystrobin concentrations: 4 mg L^–1^ (A1), 2 mg L^–1^ (A2), 1 mg L^–1^ (A3), and 0 mg L^–1^ (A4). Imidacloprid concentrations: 750 mg L^–1^ (B1), 500 mg L^–1^ (B2), 250 mg L^–1^ (B3), and 0 mg L^–1^ (A4). The mean value ± *p* < 0.05 error bar data were presented in the figure and different alphabetical letters indicate the significant differences between the different imidacloprid concentrations.

**FIGURE 11:**
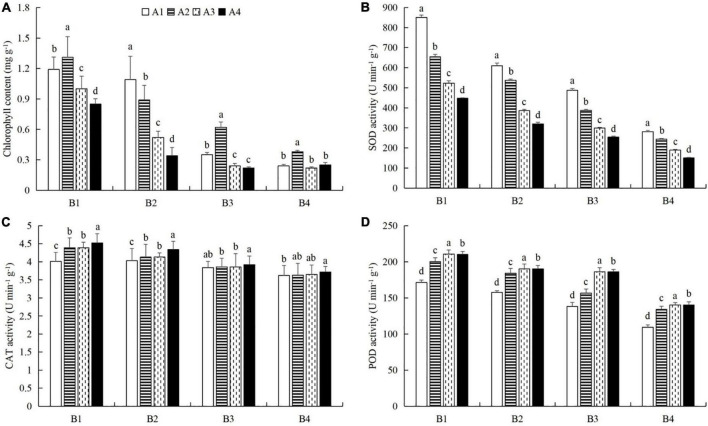
The effects of azoxystrobin on **(A)** chlorophyll content and the activities of **(B)** superoxide dismutase (SOD), **(C)** catalase (CAT), and **(D)** peroxidase (POD) in cucumber leaves at different imidacloprid concentrations. Azoxystrobin concentrations: 4 mg L^–1^ (A1), 2 mg L^–1^ (A2), 1 mg L^–1^ (A3), and 0 mg L^–1^ (A4). Imidacloprid concentrations: 750 mg L^–1^ (B1), 500 mg L^–1^ (B2), 250 mg L^–1^ (B3), and 0 mg L^–1^ (A4). The mean value ± *p* < 0.05 error bar data were presented in the figure and different alphabetical letters indicate the significant differences between the different azoxystrobin concentrations.

**FIGURE 12:**
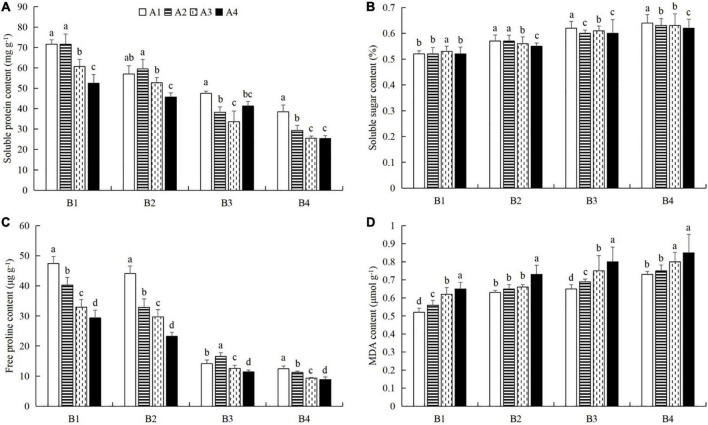
The effects of azoxystrobin on the contents of **(A)** soluble proteins, **(B)** soluble sugars, **(C)** free proline, and **(D)** malondialdehyde (MDA) in cucumber leaves at different imidacloprid concentrations. Azoxystrobin concentrations: 4 mg L^–1^ (A1), 2 mg L^–1^ (A2), 1 mg L^–1^ (A3), and 0 mg L^–1^ (A4). Imidacloprid concentrations: 750 mg L^–1^ (B1), 500 mg L^–1^ (B2), 250 mg L^–1^ (B3), and 0 mg L^–1^ (A4). The mean value ± *p* < 0.05 error bar data were presented in the figure and different alphabetical letters indicate the significant differences between the different azoxystrobin concentrations.

At a given azoxystrobin concentration, with increases in the imidacloprid concentration, we found that the chlorophyll, soluble protein, and free proline contents and the SOD, CAT, and POD activities in the leaves were increased, while the contents of soluble sugars and MDA decreased (Figures 9, 10). This indicates that imidacloprid could help the plants resist aphid feeding and powdery mildew infection by increasing the contents of chlorophyll, soluble protein, and free proline and the activities of protective enzymes and by decreasing the contents of soluble sugars and MDA in cucumber leaves. At a given imidacloprid concentration, the contents of soluble proteins, soluble sugars, and free proline increased in the leaves, as did the SOD activity, while the POD and CAT activities and the MDA content decreased with increasing azoxystrobin concentration (Figures 11, 12). These results indicated that azoxystrobin can help plants resist aphids and powdery mildew infection by increasing the contents of chlorophyll, soluble proteins, soluble sugars, and free proline, increasing the SOD activity, and decreasing soluble sugar content, POD and CAT activity, and the MDA content in cucumber leaves.

When the concentration of azoxystrobin was 750 mg L^–1^ (A1), the chlorophyll content, SOD, CAT and POD activities, and the soluble protein and free proline contents when the concentration of imidacloprid was 4 mg L^–1^ (B1) were significantly increased by 0.95 mg g^–1^ (LSD test: *p* = 0.015; Figure 9A), 569.17 U min^–1^ g^–1^ (LSD test: *p* = 0.033; Figure 9B), 0.39 U min^–1^ g^–1^ (LSD test: *p* = 0.02; Figure 9C), 61.87 U min^–1^ g^–1^ (LSD test: *p* = 0.028; Figure 9D), 33.18 mg g^–1^ (LSD test: *p* = 0.011; Figure 10A), and 34.99 μg g^–1^ (LSD test: *p* = 0.034; Figure 10C), respectively, compared with the 0 mg L^–1^ treatment (B4). The contents of soluble sugars and MDA were significantly decreased by 0.08% (LSD test: *p* = 0.019; Figure 10B) and 0.19 μmol g^–1^ (LSD test: *p* = 0.008; Figure 10D), respectively. When the concentration of imidacloprid was 4 mg L^–1^ (B1), the chlorophyll content, SOD activity, and soluble protein and free proline contents when azoxystrobin was at 750 mg L^–1^ (A1) were significantly increased by 0.34 mg g^–1^ (LSD test: *p* = 0.042; Figure 11A), 402.99 U min^–1^ g^–1^ (LSD test: *p* = 0.038; Figure 11B), 19.18 mg⋅g^–1^ (LSD test: *p* = 0.013; Figure 12A), and 18.07 μg g^–1^ (LSD test: *p* = 0.035; Figure 12C) compared with azoxystrobin at 0 mg L^–1^ (A4), respectively. The CAT and POD activities and the MDA content were significantly decreased by 0.51 U min^–1^ g^–1^ (LSD test: *p* = 0.021; Figure 11C), 38.91 U min^–1^ g^–1^ (LSD test: *p* = 0.002; Figure 11D), and 0.13 μmol g^–1^ (LSD test: *p* = 0.044; Figure 12D), respectively. The mixed use of imidacloprid and azoxystrobin can help cucumber plants resist aphid attack and powdery mildew infection more than either imidacloprid or azoxystrobin alone.

## Discussion

The results of our study showed that the physiological regulatory mechanisms that allowed cucumber plants to resist powdery mildew infection and melon aphid feeding, either alone or in combination, were different, but they all involved regulating the activities of protective enzymes. Powdery mildew infection after melon aphid infestation is mutually beneficial to both the pathogen and pest in their interaction with cucumber. However, melon aphid infestation following powdery mildew infection is unfavorable to both in their interaction with cucumber plants. The resistance of cucumber to melon aphid and powdery mildew was improved by the application of azoxystrobin and imidacloprid.

Pathogen infection and feeding by phytophagous insects can cause complex physiological and chemical changes in the host plants (Chen et al., 2019; Li et al., 2022; Naik et al., 2022). For example, the ability of plants to resist pathogen infection is enhanced by regulating photosynthesis, the synthesis of basic or secondary metabolites, and the activities of protective enzymes (Forkner et al., 2004; Chomel et al., 2016; Franco et al., 2019; Zhang Q. C. et al., 2022b). Previous studies have shown that after cucumber plants are infected by pathogens such as downy mildew and powdery mildew, they can resist the infection of pathogens by increasing the leaf chlorophyll content and enhancing photosynthesis (Zhou et al., 2013; Meng et al., 2014; Zhang H. M. et al., 2021). In addition, cucumber also regulates the synthesis of soluble sugar, soluble protein, free proline and other basic metabolites (Zhou et al., 2013; Meng et al., 2014), changes the activity of protective enzymes (Meng et al., 2012; Zhang P. et al., 2021), and reduces the content of MDA *in vivo* (Zhou et al., 2013). When cucumber plants are damaged by the leaf miner *Liriomyza huidobrensis*, the can adapt to insect stress by regulating the main nutrients, secondary metabolites, chlorophyll content, and defensive enzyme activity (Sun et al., 2012, 2013). In our previous studies, we found that cotton aphid feeding can also cause changes in the photosynthetic capacity, nutrient levels, metabolic pathways, protective enzyme activities, and other defense mechanisms in the host cotton plants (Zhang et al., 2020; Zhang Q. C. et al., 2022a,b). In this study, we found that when only infected with powdery mildew, the cucumber plants were able to resist the powdery mildew pathogen by increasing the soluble protein and soluble sugar contents, and regulation the activities of protective enzymes, but the response of protective enzymes is not so intense (Figures 1, 2A,B). In previous studies, the protective enzyme activity of cucumber in 0–9 or 0–7 days was measured, and the protective enzymes activity of cucumber increased or decreased first and then increased (Jiang and Si, 2010; Zhang H. M. et al., 2021). However, the same thing was that when the time was ≥6 days, the protective enzymes activity in cucumber was higher than that in 0 day (Jiang and Si, 2010; Zhang H. M. et al., 2021). Therefore, in the powdery mildew-cucumber system, the short-term defense effect of protective enzymes is not so obvious. And this is also related to cucumber varieties, resistant varieties of protective enzyme activity is higher than sensitive varieties (Zhang H. M. et al., 2021). In our results, although the protective enzyme activity of cucumber did not change significantly after powdery mildew infection, it was basically higher than that of healthy plants. This is consistent with the findings and phenomena of the above study (Jiang and Si, 2010; Zhang H. M. et al., 2021). At the same time, the varieties we used were sensitive to powdery mildew, which also led to the response of protective enzymes in cucumber to powdery mildew infection seems not so intense. Therefore, these reasons may lead to the fact that the protective enzyme activity in the results of this study does not appear to have a particularly significant change. When the plant were subjected to aphid feeding only, they were able to resist the aphids by increasing the protective enzyme activities and the soluble sugar content (Figures 3B–D, 4B).

However, in the actual production process, the combination of pathogen infection and pest infestation on host plants is common. In pathogen-insect-host plant systems, many studies have shown that when plants are infected by diseases, the volatile compounds released by the host plants will cause effects on insect behavior (Hulcr et al., 2011; Franco et al., 2017; González-Mas et al., 2021). This is an important defense strategy for plants to cope with the combined negative effects of pest and diseases, which avoids the superposition of the impact caused the two together, and is conducive to alleviating or extending the adaptability of plants to pathogen infection and insect attack (Ali et al., 2021; Franco et al., 2022). In addition, while plants are inevitably exposed to pests and diseases, they may adopt a more direct physiological and metabolic defense mechanism to resist pathogen infection and insect damage (Jogaiah et al., 2020; Ling et al., 2022; Zahra et al., 2022). We found that cucumber plants resisted the combination of powdery mildew infection and melon aphid feeding by increasing protective enzyme activity (Figures 5B–D). Whether powdery mildew or melon aphid acted individually or together, cucumber plants resisted pathogen infection and insect feeding by regulating the activities of protective enzymes. Therefore, our results strongly suggest that protective enzymes are an important mechanism by which cucumber plants increase their resistance to powdery mildew disease and melon aphid attack under different infection/infestation patterns.

Pathogens and insects continuously affect the growth and development of plants. Although plants have evolved complex defense strategies (Wang S. Y. et al., 2021), at the same time, pests and diseases have evolved corresponding strategies to create favorable conditions for their interaction with the host (Chang et al., 2021; Pan et al., 2021). For example, in order to better colonize the host and reproduce, pests and pathogens will choose cooperation and mutual benefit to jointly resist defense substances and physiological reactions from plants (Dáder et al., 2017; Li et al., 2017; Jayasinghe et al., 2021; Franco et al., 2022). Of course, some pests and pathogens also choose non-cooperative relationships (Noman et al., 2021; van Dijk et al., 2021). Previous studies have shown that one premise for pathogens and insects to choose cooperative or non-cooperative relationships is the order in which they infect host plants (Meng, 2013). When a pathogen first infects a plant, it will induce the plant to produce volatile substances to avoid insect attack (Ederli et al., 2021), and synergistic interactions between pathogens and insects have been utilized as a classical approach to the biological control of weeds (Willsey et al., 2017). However, when insects first attack, they often do not adversely affect the pathogens that subsequently infect, and make the host even more conducive to infection by pathogens (Li et al., 2021). For example, feeding by the willow leaf beetle *Plagiodera versicolora* induces a systemic effect in hybrid willow on subsequent infection by the rust fungus *Melampsora allii-fragilis* (Simon and Hilker, 2003). In our study, we observed another interesting phenomenon: compared with simultaneous powdery mildew infection and aphid colonization, the activities of protective enzymes in cucumber infected by powdery mildew after aphid feeding decreased (Figures 7B–D), and the effect was particularly significantly with respect to CAT activity (Figure 7C). The protective enzyme activities in cucumber colonized by aphids after powdery mildew infection increased (Figures 7B–D), especially the activities of SOD and CAT (Figure 7C). Therefore, CAT may be the key protective enzyme for cucumber to respond to the timing of powdery mildew infection and aphid attack. The changes in protective enzyme activities also indicate that powdery mildew infection after aphid colonization is mutually beneficial to both pest and pathogen (the protective enzyme activity is lower than in the simultaneous infection/colonization), but aphid colonization after powdery mildew infection is unfavorable to both (the protective enzyme activity is higher than in the combined pathogen infection and aphid colonization). Based on these results, we conclude that the sequence and timing of aphid colonization and powdery mildew infection also affect the immune response of cucumber plants.

In general, after crops become infected with diseases or are attached by insect pests, we choose to use chemical control agents to reduce the yield loss. After applying pesticides, the desired outcome is to reduce the occurrence of pests and diseases and to alleviate the stress on the plants. However, our latest research shows that mepiquat chloride (DPC), a plant growth regulator, not only has toxic effects on cotton aphids (*A. gossypii*), but also enhances the defense response of cotton plants against cotton aphids (Zhang Q. C. et al., 2022a). In this study, we also found that both azoxystrobin and imidacloprid treatment increased chlorophyll (Figures 9A, 11A), soluble protein (Figures 10A, 12A), and free proline contents (Figures 10C, 12C), and increased SOD activity (Figures 9B, 11B) and decreased MDA content (Figures 10D, 12D) in cucumber leaves. However, these compounds had opposite effects on the regulation of CAT (Figures 9D, 11D) and POD (Figures 9C, 11C) activities and the content of soluble sugars (Figures 10B, 12B). Compared with azoxystrobin or imidacloprid alone, the mixture was more conducive to improving the resistance of cucumber plants to the combination of powdery mildew infection and melon aphid feeding. This provides a new paradigm for the use of chemicals in the future. We understand that there are two ways to help plants resist pests and pathogens. One is that chemicals directly act on harmful organisms and reduce their occurrence via mechanisms of toxicity. The other way is to help plants resist the effects of harmful organisms by improving innate plant defense responses.

It is worth noting that cucumber powdery mildew and melon aphid have been resistant to many pesticides under the background of existing complex drugs (Gul et al., 2019; Nie et al., 2021). In particular, the resistance of aphids to insecticides has developed rapidly, and they have developed resistance to thiamethoxam, dinotefuran, acetamiprid, clothianidin, and so on (Chen et al., 2020; Ullah et al., 2020a,b; Zhang H. H. et al., 2022c). The cucumber powdery mildew (*Podosphaera xanthii*) was also reported resistant to boscalid, flutianil, and pyriofenone (Miyamoto et al., 2010, 2020; Ishii et al., 2011). According to our results, low doses of azoxystrobin and imidacloprid can induced cucumber defense responses to powdery mildew and melon aphid. Therefore, in the early stage of cucumber powdery mildew and melon aphid occurrence, low-dose pesticides can be used to control, and the maximum dose should not exceed the recommended dose in the field, which has a positive effect on slowing down the resistance development of cucumber powdery mildew and melon aphid to pesticides. Thus, our results provide potential management strategies for the sustainable use of pesticides and integrated pest control.

Overall, our study showed the physiological and metabolic defense mechanisms in cucumber against powdery mildew and melon aphid under different infection modes, determined the relationship between powdery mildew and melon aphid under different sequences and timing of infection/infestation, and further explored the effects of pesticides on resistance to powdery mildew infection and melon aphid colonization in cucumber. By comparing different infection/infestation modes, we found that the regulation of protective enzyme activities is the key way for cucumber plants to deal with powdery mildew disease and aphids. Interestingly, compared with simultaneous powdery mildew infection and aphid colonization, the activities of protective enzymes in cucumber infected with powdery mildew after aphid infestation decreased, especially the CAT activity. The protective enzyme activity in cucumber plants colonized by aphids after powdery mildew infection increased, especially the activities of SOD and CAT. Therefore, we think that CAT may be the key enzyme in the response of cucumber to powdery mildew infection and melon aphid colonization timing. The changes in protective enzyme activities also indicate that powdery mildew infection after aphid colonization is mutually beneficial to the pest and pathogen in their interactions with cucumber plants, but aphid colonization following powdery mildew infection is unfavorable for both. Finally, we found that the use of a fungicide and insecticide improved the resistance of cucumber plants to the combination of powdery mildew infection and aphid colonization. However, the mechanism by which cucumber plants respond to the different powdery mildew/melon aphid infection/infestation modes will require further investigation to identify the genes/proteins, pathways, and regulatory aspects involved.

## Data availability statement

The raw data of the presented results of this study are available on request to the corresponding author.

## Author contributions

QZ wrote the manuscript. MZ processed the data and drew the figures for the manuscript. JW designed and reviewed the manuscript. All authors have read and approved the final manuscript.
